# Mutation of PXR phosphorylation motif at Ser347 disrupts lipid and bile acid homeostasis in diet-induced metabolic dysfunction–associated steatohepatitis in mice

**DOI:** 10.1016/j.dmd.2025.100222

**Published:** 2025-12-22

**Authors:** Veronia Basaly, Zakiyah R. Henry, Rulaiha E. Taylor, Bo Kong, Ill Yang, Anita Brinker, Zhenning Yang, Peihong Zhou, Laurie B. Joseph, Lauren Aleksunes, Brian Buckley, Masahiko Negishi, Grace L. Guo

**Affiliations:** 1 Department of Pharmacology and Toxicology, Ernest Mario School of Pharmacy, Rutgers University, Piscataway, New Jersey; 2 Environmental and Occupational Health Science Institute, Rutgers University, Piscataway, New Jersey; 3 Rutgers Center for Lipid Research, Rutgers University, New Brunswick, New Jersey; 4 Reproductive and Developmental Biology Laboratory, National Institute of Environmental Health Sciences, NIH, Chapel Hill, North Carolina; 5 Department of Veterans Affairs New Jersey Health Care System, East Orange, New Jersey

**Keywords:** PXR, Phosphorylation, PXR-KI, Metabolic dysfunction–associated steatohepatitis, Lipogenesis, Bile acids

## Abstract

The pregnane X receptor (PXR), a ligand-activated transcription factor, regulates the expression of genes involved in endobiotic and xenobiotic metabolism, inflammation, and fibrosis. Disruption of PXR functions can affect processes critical to metabolic dysfunction–associated steatohepatitis (MASH) progression. Although ligand-dependent PXR functions are well studied, its regulation by post-translational modification, particularly phosphorylation, remains unclear. PXR has a conserved phosphorylation motif within its ligand binding domain (Ser347 in mice; Ser350 in humans). In vitro studies showed that this site mutation impairs human PXR transcriptional activity; however, the mechanism remains elusive. To investigate this phosphorylation site role in MASH development, wild-type and PXR Ser347Ala knock-in mutation (PXR-KI) mice were fed either a high-fat diet or a control chow diet for 16 weeks. On control chow diet, PXR-KI mice exhibited decreased expression of alternative bile acid (BA) synthesis genes compared with wild-type mice. On a high-fat diet, PXR-KI mice manifested more severe hepatic steatosis, revealed by elevated serum total cholesterol, and increased expression of genes involved in lipid metabolism. In addition, changes in BA metabolism and transporter genes suggested a cholestatic pattern in this group of mice. BA profiling showed higher levels of conjugated, hydrophilic, primary BA in the serum and liver, and increased unconjugated BA in the intestine. The data suggest that PXR Ser347 phosphorylation motif is essential for regulating PXR functions to maintain endobiotic metabolism and alleviate hepatotoxicity during MASH progression.

**Significant Statement:**

The ligand-independent role of pregnane X receptor (PXR) is unclear. In phosphodeficient PXR knock-in mice, loss of Ser347 phosphorylation worsened hepatic steatosis and altered bile acid homeostasis under high-fat diet feeding, uncovering a novel role and therapeutic potential of PXR phosphorylation in fatty liver diseases.

## 1 Introduction

Metabolic dysfunction–associated steatotic liver disease is the most prevalent chronic liver disease,^1^ affecting approximately 32% of adults worldwide.^2^^,^^3^ Metabolic dysfunction–associated steatotic liver disease is characterized by liver steatosis, which can progress to metabolic dysfunction–associated steatohepatitis (MASH). MASH is characterized by severe steatosis, hepatocyte ballooning, and inflammation, and often accompanied by fibrosis, which can further progress to cirrhosis or even hepatocellular carcinoma.^4^ The pathogenesis of MASH is complex, involving factors such as obesity, mitochondrial dysfunction, oxidative stress, and genetic and epigenetic factors. All these factors act together and result in hepatic lipid accumulation, cellular injury, and activation of hepatic stellate cells, which eventually leads to fibrosis.^5^

The pregnane X receptor (PXR; NRI2) is a nuclear receptor responsible for regulating the expression of genes critical for drug-metabolizing enzymes and transporters.^6^ Also, PXR is essential for endobiotic metabolism and energy homeostasis by regulating genes involved in glucose, lipid, and bile acid (BA) metabolism, as well as inflammation and fibrosis.^7^ PXR contributes to the regulation of various liver functions and pathways; therefore, dysregulation of these PXR-mediated pathways is implicated in MASH pathogenesis. There is an urgent need to understand the molecular mechanisms related to PXR regulation of endobiotic metabolism and energy homeostasis for the discovery of new interventions for therapeutic agents.

PXR is well known as a ligand-dependent xenobiotic sensor and serves as a physiological sensor in diverse cellular processes. Several studies indicated that ligand activation of PXR can promote lipogenesis,^8^, ^9^, ^10^ reduce gluconeogenesis,^11^, ^12^, ^13^ and exert an anti-inflammatory and antifibrotic effect.^6^ However, the molecular mechanisms and pathways related to PXR regulation of its hepatic functions in a ligand-independent manner through post-translational modification, such as phosphorylation, remain elusive. Several studies revealed that mutation at multiple serine and threonine phosphorylation residues alters PXR activity by affecting subcellular localization, decreasing heterodimerization ability with retinoid X receptor, reducing interaction with the coactivator, steroid receptor coactivator-1, and abolishing ligand-induced PXR activity. These mutations were generated by changing phosphorylated serine into phosphomimetic glutamate or aspartate or nonphosphorylated alanine.^14^ PXR has a conserved phosphorylation motif in its ligand binding domain, Ser347 in mouse PXR and Ser350 in human PXR, that can regulate PXR-retinoid X receptor heterodimerization.^15^ Some studies focused on studying the effects of Ser350 in regulating PXR transcriptional activity, and the results showed that phosphomimetic mutation of PXR S350 (S350D) attenuates PXR transcription activity, whereas the phosphodeficient mutant Ser350 (S350A) shows partial resistance to the inhibitory effect of CDK2 kinases.^16^ However, very little is known about the effect of blocking Ser347/350 on PXR regulation of endobiotic metabolism and energy homeostasis.

Interestingly, low glucose levels stimulate PXR phosphorylation at Ser350 to induce gluconeogenesis by stimulating serum/glucocorticoid regulated kinase 2 dephosphorylation.^17^ Also, studies using a PXR phosphorylation-blocking at Ser347 (PXR Ser347Ala knock-in, PXR-KI) mouse model reported that fasting PXR-KI mice developed hypertriglyceridemia and increased hepatic lipid and triglyceride levels.^18^ These findings indicate that PXR Ser347 phosphorylation motif plays a role in maintaining hepatic energy homeostasis during fasting in mice or under low glucose conditions. The role of PXR phosphorylation at Ser347 in regulating endobiotic metabolism during MASH development has not been previously evaluated. The objective of this study was to investigate the effects of blocking PXR at Ser347 on MASH disease progression using a PXR-KI mouse model. In this study, we aim to shed light on the impact of phosphorylation at Ser347 in regulating ligand-independent PXR-mediated pathways involved in maintaining energy homeostasis and MASH development. In addition, we seek to explore the role of physiological pathways in regulating PXR hepatic functions to uncover underlying mechanisms for future therapeutic strategies to treat/prevent MASH progression.

## 2 Materials and methods

### 2.1 Animals and treatments

Six-week-old male wild-type (WT) and PXR-KI mice, both on the C57BL/6J genetic background,^18^ were fed either a high-fat diet (HFD) that provided 40 Kcal% palm oil fat, 20 Kcal% fructose, and 2% cholesterol (D09100310; Research Diets), or a control chow diet (CCD; 5053 PicoLab Rodent Diet 20; LabDiet 20) containing approximately 20% protein and 4.5% fat for 16 weeks. Mice were grouped as WT mice on a CCD (*n* = 3), WT mice on an HFD (*n* = 5), PXR-KI mice on a CCD (*n* = 5), and PXR-KI mice on an HFD (*n* = 8). Body weights (BWs) were monitored and recorded weekly. All animals were housed and maintained under standard laboratory conditions with 12-hour light/dark cycles. Food and water were provided ad libitum. Animals were euthanized by the end of the experiment, and tissues (blood, liver, and small intestine) were collected for further analysis. In this study, all animal experiments were approved by the Rutgers Institutional Animal Care and Use Committee.

### 2.2 Glucose tolerance test

At week 12 of 16 weeks of HFD feeding, an oral glucose tolerance test was performed. Mice were fasted overnight for approximately 12 hours. Blood glucose levels were measured before and after the administration of 2 g/kg BW of glucose solution (20% d-glucose solution, W:V, dissolving 2 g of glucose in 10 mL of saline) to mice via oral gavage. Blood from the tail vein was used to detect the blood glucose levels at multiple time points (0, 15, 30, 60, and 120 minutes) using a glucometer. The area under the curve (AUC) was calculated to assess the glucose tolerance of each group.

### 2.3 Serum biochemistry

Activities of serum alanine aminotransferase and alkaline phosphatase, as well as concentrations of total cholesterol, triglyceride, glucose, albumin, and bilirubin, were measured using the Heska Element DC5X Blood Chemistry Analyzer.

### 2.4 Gene expression at mRNA level

Total RNA was extracted from snap-frozen ileum and liver samples using TRIzol reagent (Thermo Fisher Scientific). The complementary DNA was obtained by reverse transcription. The relative gene expression was quantified using the Viia7 real-time quantitative polymerase chain reaction machine (Life Technologies) with SYBR Green chemistry in a 384-well plate format. Each reaction was prepared in a total of 8.0 *μ*L per well, containing 4.0 *μ*L of 2× SYBR Green Master Mix, 0.1 *μ*L of primer mix (20 *μ*M), 1.9 *μ*L of sterile water, and 2.0 *μ*L of diluted cDNA. Polymerase chain reaction amplification was conducted under the following standard conditions: an initial denaturation at 95 °C for 10 minutes, followed by 40 cycles of 95 °C for 15 seconds and 60 °C for 60 seconds. All Ct values were converted to ΔΔCt values and normalized to the mRNA levels of a housekeeping gene, Gapdh. Sequences of primers used can be found in Supplemental Table 1.

### 2.5 Liver lipid concentration measurement

Cholesterol and triglycerides were extracted from snap-frozen liver tissues (50–100 mg) via homogenization with lipid extraction buffer (18 mM Tris, pH 7.5, 300 mM mannitol, 50 mM EGTA, and 0.1 mM phenylmethylsulfonyl fluoride). The homogenate (400 *μ*L) was mixed with 2 mL of chloroform-methanol (2:1) solution in glass tubes and rocked overnight at room temperature. Water (1 mL) was added, and the mixture was vortexed and centrifuged for 5 minutes at 3000*g* to produce a biphasic system. Pure lipid extract was obtained by pipetting the lower layer (2 mL) into a new glass tube and was dried using SpeedVac (Thermo Fisher Scientific). The lipid pellets were dissolved in a mixture of 60 *μ*L of *tert*-butanol and 40 *μ*L of Triton X-114: methanol (2:1) mix. Triglyceride and cholesterol levels were measured using commercially available lipid assay kits (Pointe Scientific) and spectrophotometry. Calculated lipid levels were normalized to the amount of liver tissue used, and data were reported as milligrams of lipid per gram of liver weight (LW).

### 2.6 Histopathology and immunohistochemical analyses

Liver samples were fixed in 10% PBS neutral buffered formalin and embedded in paraffin wax. Liver sections were cut at 4 *μ*m thickness and stained with H&E for general histologic feature assessment (*n* = 3–8). Immunohistochemistry was conducted on liver sections to detect the macrophage activation protein expression using an antibody against rat F4/80 (F4/80; 1:1000; Biorad) or IgG control as previously described.^19^ Tissue sections were scanned using an Olympus VS120 microscope. Semiquantitative analyses were performed using percent positive stain on 5 representative images per animal per treatment group. Images were quantified at 10× magnification using the Fiji package of ImageJ. The antibodies used are listed in Supplemental Table 2. Fast Green-Sirius Red (FGSR) stained liver sections were used to measure collagen deposition. FGSR was prepared using 0.1% (w/v) Direct Red 80 (cat. 0-0303; Sigma) and 0.1% (w/v) Fast Green FCF (cat. F-00; Thermo Fisher Scientific) in saturated aqueous picric acid (cat. P6744; Sigma).

### 2.7 BA extraction and liquid chromatography-tandem mass spectrometry profiling

Total BAs were extracted and purified from all treatment groups for the serum, liver, and small intestine. Tissue homogenization was performed on the liver and small intestine using a ratio of 55 mg of tissue in 300 *μ*L of high-performance liquid chromatography grade H_2_O. To perform BA extraction, 45 *μ*L of serum or 300 *μ*L of liver or whole small intestine homogenate was added to 450 *μ*L or 900 *μ*L of cold methanol, for serum and liver/small intestine tissue homogenate, respectively. Samples were coincubated with internal standard BA mixture (*β*-muricholic acid-d4, cholic acid-d4, and chenodeoxycholic acid-d4) for 1 hour at room temperature on a shaker table and then centrifuged at 12,000*g* for 15 minutes. For serum, supernatant was collected and filtered through a 0.22-*μ*m Costar Spin-X centrifuge filter tube. For the liver and small intestine, supernatant was collected, and sample pellets were resuspended in 600 *μ*L of 50% methanol, recentrifuged, and the supernatant was collected, combined with the first collection, and filtered through a 0.1-*μ*m Costar Spin-X centrifuge filter tube. The filtrate was used for analysis. All BA extracts were analyzed using a Thermo Accela Ultra Performance Liquid Chromatography System (Thermo Fisher Scientific) coupled with a Thermo Finnigan LTQ XL ion trap mass spectrometer. Chromatography was performed using a reverse-phase C18 Thermo Hypersil GOLD column (2.1 × 50 mm, 1.9 *μ*m particle size). The mobile phase consisted of (A) 0.15% formic acid in methanol, (B) 0.10% formic acid in water, and (C) 100% acetonitrile. The gradient program was as follows: started 10% A, 70% B, 20% C until 0.5 minutes, reached 15% A, 55% B, 30% C at 5 minutes, 20% A, 40% B, 40% C at 7 minutes, 30% A, 30% B, 40% C at 8.5 minutes, 40% A, 15% B, 45% C at 10.5 minutes, 40% A, 10% B, 50% C at 11 minutes 40% A, 10% B, 50% C at 11 minutes, 5% A, 2% B, 93% C at 11.5 minutes, 0% A, 3% B, 97% C at 14.5 minutes, and then back to the initial condition. The flow rate was 310 *μ*L/min, the sample injection volume was 5 *μ*L, and the column temperature was maintained at 25 °C. The LTQ XL was operated in negative electrospray ionization with a selective ion mode after the manufacturer’s mass calibration and tuning procedure. Electrospray ionization parameters include 4.4 kV ion spray voltage, 300 °C of capillary temperature, 42 V of capillary voltage, 100 V of tube lens voltage, 37 arb of nitrogen sheath gas flow rate, and 13 arb Aux nitrogen gas flow rate with no sweep gas flow. Data processing and quantification were performed using Xcalibur quantification software (Thermo Fisher Scientific). Calculations of BA indices were performed as described by Alamoudi et al.^20^

### 2.8 Statistical analyses

Data are presented as mean ± SD. GraphPad Prism (version 10; GraphPad Software Inc) was used for statistical analysis. Two-way ANOVA was performed, followed by Tukey’s multiple comparison test to evaluate differences among individual groups. Differences were considered statistically significant at *P* < .05.

### 2.9 Graphics and illustrations

All data plotting and graphical illustration were created using GraphPad Prism Software version 10 (GraphPad Software Inc). All scientific illustrations were created with Biorender Software.

## 3 Results

### 3.1 Phenotypic characterization of PXR-KI mice on HFD

**Fig. 1:**
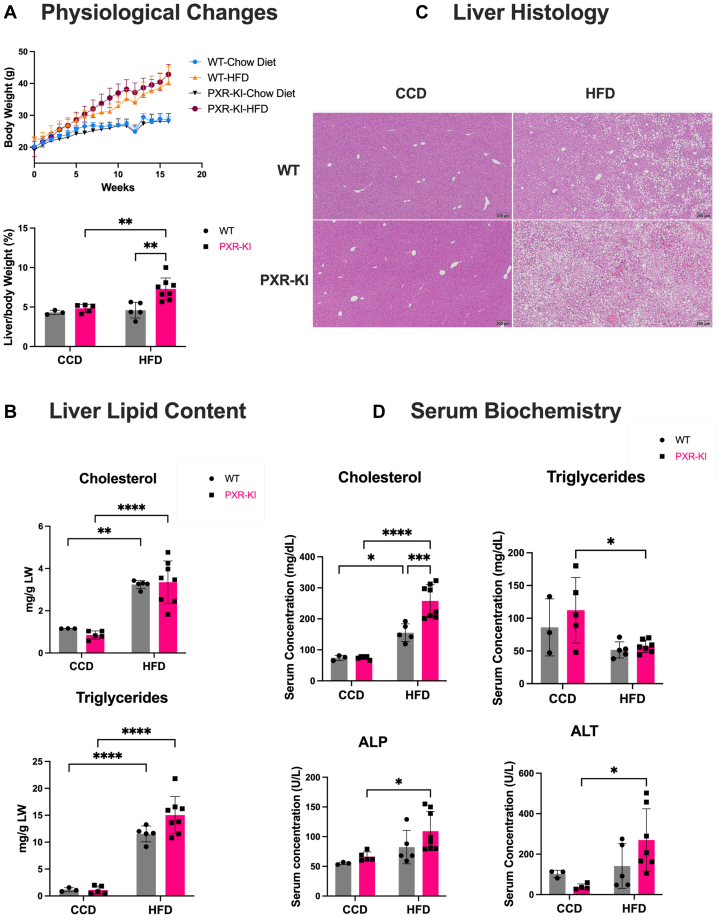
Phenotype characterization at the end of 16 weeks on diets for PXR-KI mice. (A) Male body weight change over the course of 16 weeks of feeding (top panel) and liver-to-body weight ratio (%; bottom panel). (B) Hepatic levels of total cholesterol (top panel) and triglycerides (bottom panel). (C) H&E-stained liver sections. (D) Serum biochemistry: ALT, ALP (bottom panel), and serum levels of triglycerides, and total cholesterol (top panel). Data represented as mean ± SD (*n* = 3–8). Two-way ANOVA. ∗*P* < .05; ∗∗*P* < .01; ∗∗∗*P* < .001; ∗∗∗∗*P* < .0001. ALP, alkaline phosphatase; ALT, alanine aminotransferase; CCD, control chow diet; HFD, high-fat diet; LW, liver weight; PXR-KI, pregnane X receptor Ser347Ala knock-in mutation; WT, wild-type.

On control diet, no phenotypic differences were observed between PXR-KI and WT mice in BW, liver-to-BW ratio, liver histology, serum biochemistry, or hepatic lipid content. PXR-KI mice on HFD showed an increase in LW/BW% compared with WT mice on HFD and PXR-KI mice on CCD (Fig. 1A). To assess lipid content, hepatic lipid extraction was conducted. The hepatic cholesterol and triglyceride levels were increased in PXR-KI and WT mice on HFD compared with PXR-KI and WT CCD groups, respectively. PXR-KI mice on HFD displayed a slightly higher mean level of hepatic triglycerides than WT mice on HFD; however, the difference was not statistically significant (Fig. 1B). The liver histology is consistent with LW/BW and hepatic lipid content data because PXR-KI mice on HFD had more severe liver steatosis compared with WT mice on HFD (Fig. 1C). To further assess the effect of PXR-KI on MASH development, after 16 weeks of feeding, serum biochemistry was analyzed. PXR-KI mice on HFD displayed an increase in serum total cholesterol levels compared with all other groups. The serum triglyceride levels were decreased in PXR-KI mice on HFD compared with PXR-KI mice on CCD (Fig. 1D). The serum levels of alanine aminotransferase and alkaline phosphatase increased in PXR-KI mice on HFD compared with PXR-KI mice on CCD, and the mean levels were slightly higher (not significant) than WT mice on HFD (Fig. 1D). The serum glucose levels were elevated in PXR-KI mice on HFD versus PXR-KI mice on CCD. Compared with WT mice on HFD, no changes were observed in PXR-KI mice on HFD for serum albumin, total bilirubin, and glucose (Supplemental Fig. 1B). After 12 weeks of feeding, an oral glucose tolerance test was performed. The AUC was calculated, and the results demonstrated that WT and PXR-KI mice on HFD had an increase in AUC compared with the WT and PXR-KI CCD groups, respectively. No changes were observed between PXR-KI mice and WT mice on HFD (Supplemental Fig. 1A).

### 3.2 Effect of PXR Ser347A on MASH development

**Fig. 2:**
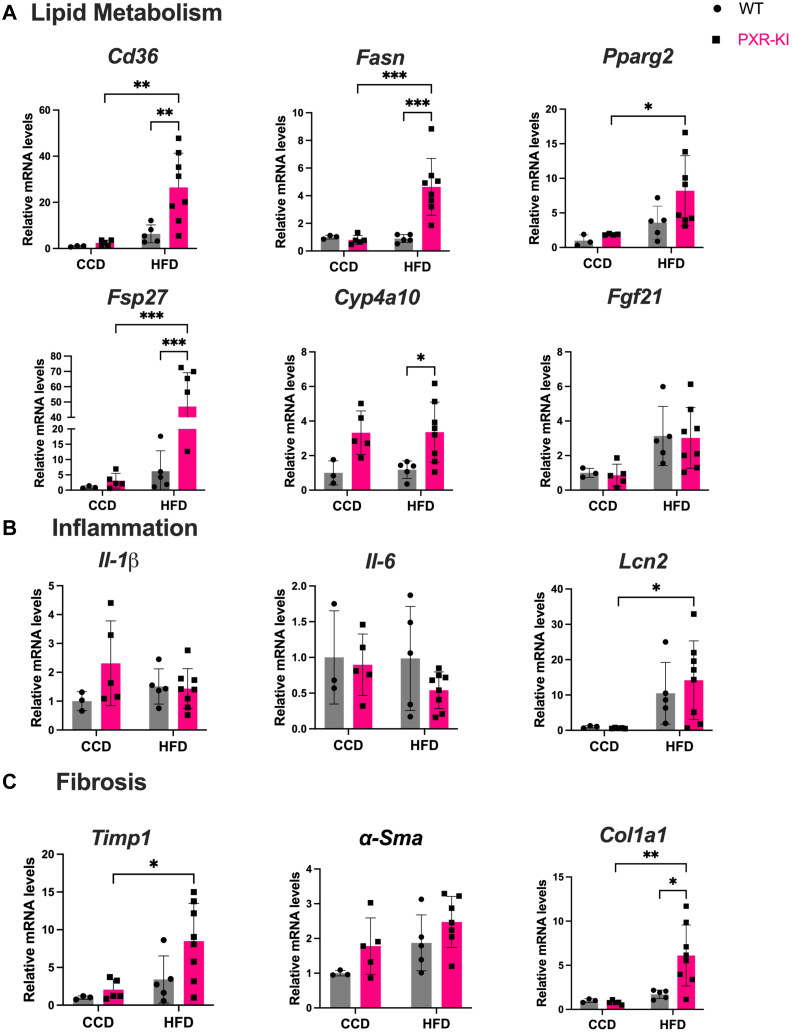
Relative mRNA quantification of genes involved in lipid metabolism, inflammation, and fibrosis. (A) Relative mRNA levels of lipid metabolism genes (*Cd36*, *Fasn*, *Pparg2*, *Fsp27*, *Cyp4a10*, and *Fgf21*). (B) Inflammation gene mRNA expression (*Il-1β*, *Il-*6, and *Lcn2*). (C) Fibrosis gene mRNA expression (*Timp1*, *αSma*, and *Col1a1*). Data are represented as mean ± SD (*n* = 3–8). Two-way ANOVA. ∗*P* < .05; ∗∗*P* < .01; ∗∗∗*P* < .001; ∗∗∗∗*P* < .0001. CCD, control chow diet; HFD, high-fat diet; PXR-KI, pregnane X receptor Ser347Ala knock-in mutation; WT, wild-type.

To assess the effect of blocking PXR phosphorylation at Ser347 on MASH development, the mRNA levels of genes involved in lipid metabolism, inflammation, and fibrosis were measured. The fatty acid translocase (*Cd36*) is the gene encoding for the long chain fatty acid transporter. PXR-KI mice on HFD showed an increase in *Cd36* mRNA levels compared with WT mice on HFD and PXR-KI mice on CCD (Fig. 2A). Similarly, the expression of fatty acid synthase (*Fasn*), a gene involved in de novo lipogenesis, was found to be increased in PXR-KI mice on HFD compared with both PXR-KI on CCD and WT on HFD groups (Fig. 2A). The peroxisome proliferator-activated receptor *γ* (PPAR*γ*) is responsible for activating the expression of genes involved in lipid and triglyceride accumulation. The fat-specific protein 27 gene (*Fsp27*), one of PPAR*γ* target genes, is also involved in lipid droplet accumulation. The mRNA levels of *Pparg2* were increased in PXR-KI mice on HFD compared with PXR-KI mice on CCD. Interestingly, PRX-KI mice on HFD displayed an increase in the mRNA levels of *Fsp27* compared with PXR-KI mice on CCD and WT mice on HFD (Fig. 2A). *Cyp4a10* and *Fgf21* are genes involved in fatty acid *β*-oxidation. The mRNA levels of *Cyp4a10* were increased in PXR-KI mice on HFD compared with WT mice on HFD. No changes were observed in mRNA levels of *Fgf21* (Fig. 2A). Interestingly, the mRNA levels of lipocalin-13 (*Lcn13*), a farnesoid X receptor (FXR) target gene, were reduced in PXR-KI mice on CCD and in WT mice on HFD compared with WT mice on CCD. PXR-KI mice on HFD exhibited the lowest expression for *Lcn13* (Supplemental Fig. 2C). For glucose metabolism, the expression of *G6Pase* and *Pepck* were decreased in PXR-KI mice on CCD compared with WT mice on CCD and in PXR-KI mice on HFD compared with WT mice on HFD. The mRNA levels of *G6Pase* were also reduced in PXR-KI mice on HFD compared with WT mice on HFD and PXR-KI mice on CCD (Supplemental Fig. 2B). For PXR target genes, the PXR-KI CCD group showed an increase in *Cyp2b10* expression compared with WT mice on CCD. Both *Cyp2b10* and *Cyp3a11* expression were decreased in PXR-KI mice on HFD compared with PXR-KI mice on CCD. *Cyp3a11* expression was reduced in WT mice on HFD compared with WT mice on CCD. No changes were observed in *Cyp2b9* expression across the treatment groups (Supplemental Fig. 2A).

We next assessed the effect of phosphorylation mutation of PXR at Ser347 on inflammation and fibrosis. No changes were observed in mRNA levels of Il-1*β*, Il-6, and *α-Sma* across the treatment groups. The mRNA levels of lipocalin-2 (*Lcn2*), a marker of inflammation, was increased in PXR-KI mice on HFD compared with PXR-KI mice CCD and showed slightly higher mean levels compared with WT mice on HFD (Fig. 2B). In addition, the mRNA levels of collagen type I alpha 1 (*Col1a1*) and tissue metalloproteinases inhibitor (*Timp1*), markers for fibrosis, were increased in PXR-KI mice on HFD compared with the PXR-KI CCD group. PXR-KI mice on HFD demonstrated an increase in mRNA levels of *Col1a1* compared with WT mice on HFD (Fig. 2C). No difference was seen across the treatment groups for the FGSR of collagen deposition and fibrosis (Supplemental Fig. 3B). Moreover, the immunohistochemistry staining of F4/80 protein, a marker for macrophage activation for inflammation, did not show any changes across the treatment groups (Supplemental Fig. 3A).

### 3.3 Effect of PXR Ser347A on BA homeostasis during MASH development

**Fig. 3:**
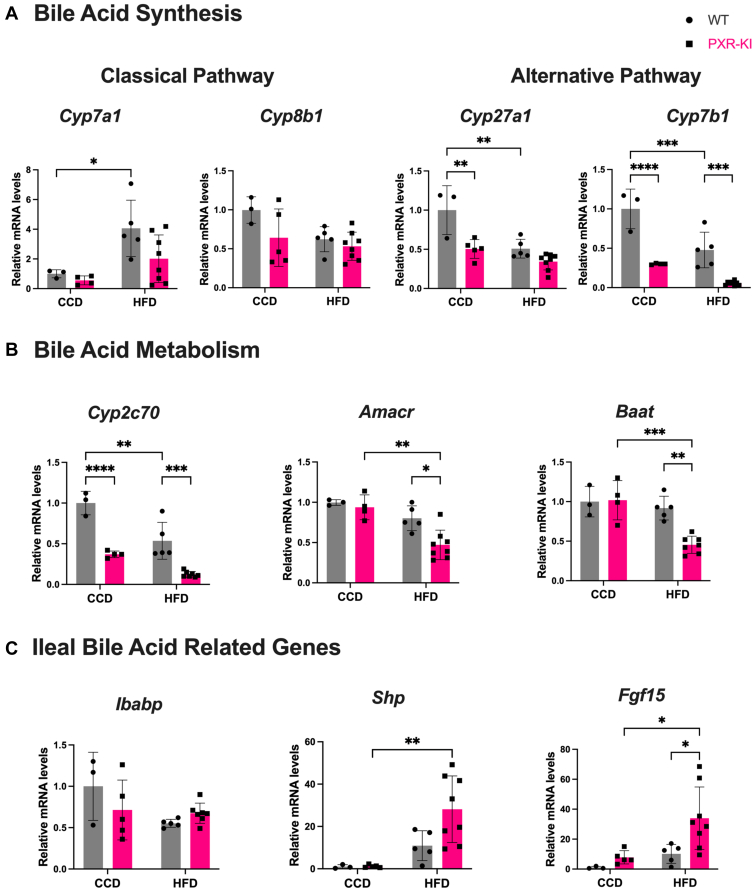
Relative mRNA quantification of BA-related genes. (A) mRNA expression of hepatic BA synthesis enzymes in classical (*Cyp7a1* and *Cyp8b1*) and alternative (*Cyp27a1* and *Cyp7b1*) pathways. (B) mRNA expression of BA metabolism/conjugation genes (*Cyp2c70*, *Amacr*, and *Baat*). (C) mRNA expression of ileal BA-related genes (*Ibabp*, *Shp*, and *Fgf15*). Data are represented as mean ± SD (*n* = 3–8). Two-way ANOVA. ∗*P* < .05; ∗∗*P* < .01; ∗∗∗*P* < .001; ∗∗∗∗*P* < .0001. BA, bile acid; CCD, control chow diet; HFD, high-fat diet; PXR-KI, pregnane X receptor Ser347Ala knock-in mutation; WT, wild-type.

**Fig. 4:**
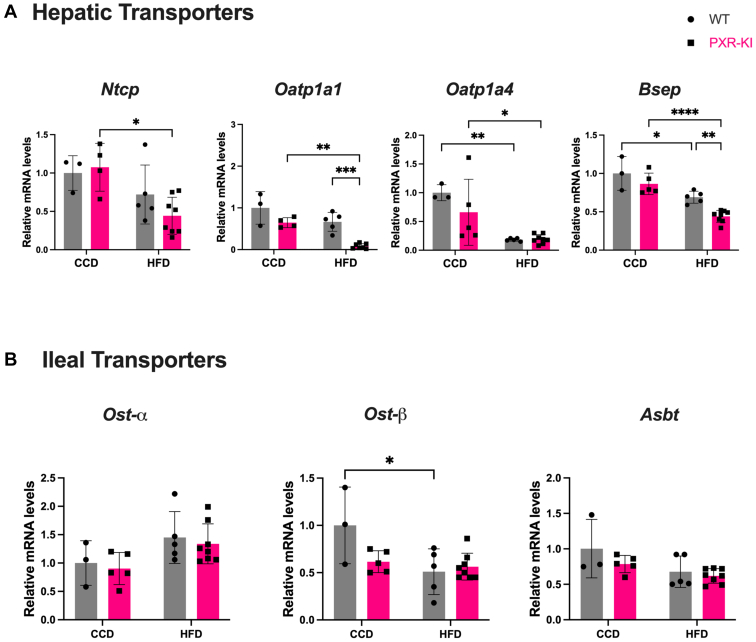
Relative mRNA quantification of hepatic and ileal BA-related transporters genes. (A) mRNA expression of hepatic BA-related transporters (*Ntcp*, *Oatp1a1*, *Oatp1a4*, and *Bsep*). (B) mRNA expression of ileal BA-related transporters (*Ost-α*, *Ost-β*, and *Asbt*). Data are represented as mean ± SD (*n* = 3–8). Two-way ANOVA. ∗*P* < .05; ∗∗*P* < .01; ∗∗∗*P* < .001; ∗∗∗∗*P* < .0001. BA, bile acid; CCD, control chow diet; HFD, high-fat diet; PXR-KI, pregnane X receptor Ser347Ala knock-in mutation; WT, wild-type.

Disruption of BA homeostasis has been associated with MASH development.^21^ To understand the effect of blocking PXR S347 phosphorylation site on BA hemostasis, we measured the relative mRNA expression of genes involved in BA metabolism and signaling pathways. The mRNA levels of cholesterol-7*α*-hydroxylase encoded by *cyp7a1*, involved in the classical synthesis pathway of BAs, were increased in WT mice on HFD compared with the WT CCD group. PXR-KI mice on HFD showed a slight reduction in the mean mRNA levels of *Cyp7a1*. No changes were observed across all groups in *Cyp8b1* expression, which encodes sterol 12-*α*-hydroxylase enzyme involved in the classical BA synthesis pathway (Fig. 3A). Interestingly, the basal mRNA levels of genes in alternative pathways, sterol-27-hydroxylase encoded by *Cyp27a1* and 25-hydroxycholesterol 7-*α*-hydroxylase encoded by *Cyp7b1,* were reduced in PXR-KI mice on CCD compared with WT mice on CCD. The expression of both *Cyp27a1* and *Cyp7b1* were decreased in WT on HFD compared with WT on CCD (Fig. 3A). A similar trend of *Cyp7b1* expression was observed for cytochrome P450, family 2, subfamily c, polypeptide 70 (*Cyp2c70*), which encodes for the enzyme that converts chenodeoxycholic acid (CDCA) into the more hydrophilic BA muricholic BA (MCA) in mice. The mRNA levels of *Cyp2c70* were decreased in PXR-KI mice on CCD and PXR-KI mice on HFD compared with the WT CCD and WT HFD groups, respectively. In addition, *Cyp2c70* expression was reduced in the WT HFD group compared with the WT CCD group. Overall, PXR-KI mice on HFD showed reduced expression of *Cyp7b1* and *Cyp2c70* compared with all other groups (Fig. 3, A and B). The *α*-methylacyl-CoA racemase encoded by *Amacr* is responsible for the conversion of BA stereoisomers, which is subsequently followed by *β*-oxidation during primary BA synthesis. The *Baat* gene encodes BA-CoA:amino acid *N*-acyltransferase, the enzyme responsible for BA conjugation. The mRNA levels of both *Amacr* and *Baat* were decreased in PXR-KI mice on HFD compared with both WT HFD and PXR-KI CCD groups (Fig. 3B). In mice, the ileal fibroblast growth factor 15 (FGF15) regulates BA synthesis through a negative feedback inhibition of hepatic expression of CYP7A1 and CYP8B1 enzymes. We then further measured the expression of ileal FXR target genes, *Fgf15* and small heterodimer partner (*Shp*). PXR-KI mice on HFD demonstrated an increase in *Fgf15* expression compared with the WT HFD and PXR-KI CCD groups. *Shp* expression was increased in PXR-KI mice on HFD compared with PXR-KI on CCD. No changes were observed in the intestinal BA binding protein (*Ibabp*) expression across the groups (Fig. 3C). We also measured the expression of hepatic BA influx and export transporters. The mRNA levels of bile salt export pump (*Bsep*) were reduced in the WT HFD and PXR-KI HFD groups compared with the WT CCD and PXR-KI CCD groups, respectively. Also, *Besp* expression was reduced in PXR-KI mice on HFD compared with WT mice on HFD (Fig. 4A). The mRNA levels of BA uptake transporters sodium taurocholate cotransporting peptide (*Ntcp*) and organic anion–transporting polypeptide (*Oatp1a1*) were decreased in PXR-KI mice on HFD compared with the PXR-KI CCD group (Fig. 4A). In addition, the expression of the uptake transporter *Oatp1a4* was decreased in both PXR-KI and WT mice on HFD compared with their counterparts, controls on CCD (Fig. 4A). Overall, PXR-KI mice on HFD showed the lowest gene expression of *Oatp1a1* compared with all groups. No significant changes were observed in ileal BA-related transporters or other hepatic transporters (Fig. 4B and Supplemental Fig. 4).

### 3.4 BA profiling

**Fig. 5:**
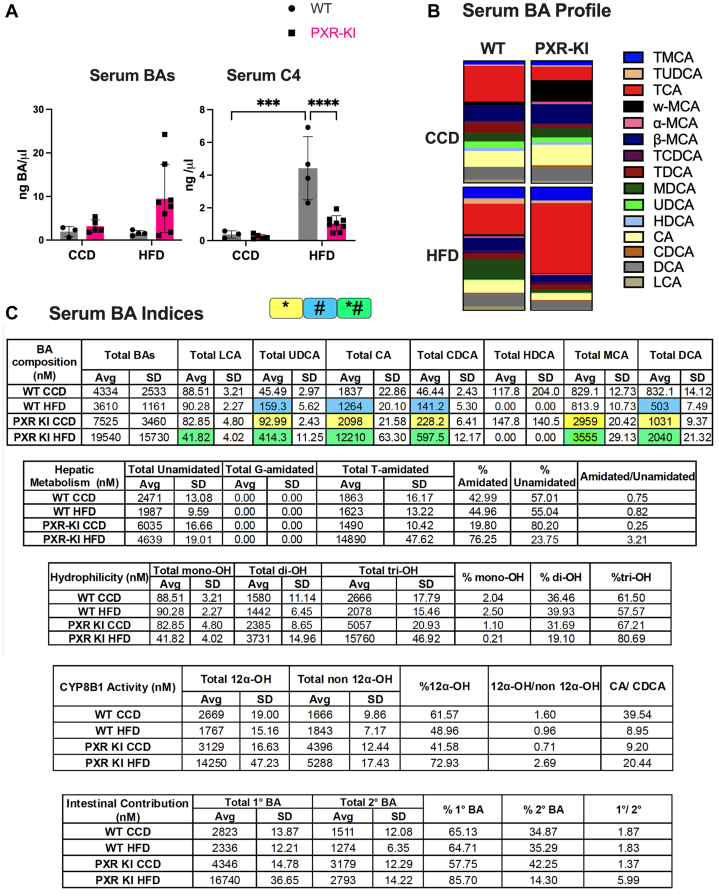
Serum BA profiles and indices. (A) Serum BA and C4 concentrations. (B) Serum BA individual species as the percentage of total BA pool per treatment group. (C) Serum BA indices calculations for the following criteria: BA composition, hepatic metabolism, hydrophilicity, Cyp8b1 activity, and intestinal absorption. Data in the table are presented as mean ± SD (*n* = 3–8). Two-way ANOVA. ∗ represents the significant difference between genotypes within the same diet, # represents the significant difference between diets within the same genotype, and ∗# represents the significant difference across both genotypes and diets. BA, bile acid; CA, cholic acid; CCD, control chow diet; CDCA, chenodeoxycholic acid; DCA, deoxycholic acid; GCA, glychcholic acid; HDCA, hyodeoxycholic acid; HFD, high-fat diet; LCA, lithocholic acid; MCA, muricholic acid; MDCA, murideooxycholic acid; PXR-KI, pregnane X receptor Ser347Ala knock-in mutation; TCA, taurocholic acid; TCDCA, taurochenodeoxycholic acid; TDCA, taurodeoxycholic acid; TLCA, taurolithocholic acid; TMCA, tauromuricholic acid; TUDCA, tauroursodeoxycholic acid; UDCA, ursodeoxycholic acid; WT, wild-type.

BA profile in the serum, liver, and small intestine has been analyzed to understand the impact of Ser347A phosphorylation mutation of PXR on a BA pool. In serum, the total BA concentration was elevated, while the C4 concentration, a marker of CYP7A1 activity, was decreased in PXR-KI mice compared with WT mice on HFD. The concentration of C4 was increased in WT mice on HFD compared with WT mice on CCD (Fig. 5A). In WT mice, compared with CCD, HFD feeding resulted in a serum BA pool showing higher percentages of tauro-muricholic acid (TMCA, 9.09%), tauroursodeoxycholic acid (TUDCA, 4.41%), and murideoxycholic acid (MDCA, 17.05%), but reduced taurodeoxycholic acid (TDCA, 3.85%). In PXR-KI mice, compared with CCD, HFD feeding resulted in a more conjugated and hydrophilic serum BA pool with higher levels of primary BAs. The serum BA pool of PXR-KI mice on HFD compared with PXR-KI mice on CCD showed higher percentages of TMCA (10.90%) and taurocholic acid (TCA, 57.16%), but reduced *ω*-MCA (0.82%), *α*-MCA (0.78%), *β*-MCA (5.68%), ursodeoxycholic acid (UDCA, 0.66%), and cholic acid (CA) (5.33%). Compared with WT mice on HFD, PXR-KI mice on HFD revealed a serum BA pool with more TCA (57.16%), but reduced CDCA (0.69%), CA (5.33%), MDCA (2.82%), and TUDCA (2.12%) (Fig. 5B). Among all other groups, PXR-KI mice on HFD displayed the highest levels of total UDCA, CDCA, and deoxycholic acid (DCA) (Fig. 5C). The CYP8B1 activity was measured based on the 12*α*-OH/non–12*α*-OH ratio. PXR-KI mice on HFD showed a higher 12*α*-OH/non–12*α*-OH ratio compared with the PXR-KI CCD and WT HFD groups (Fig. 5C).

**Fig. 6:**
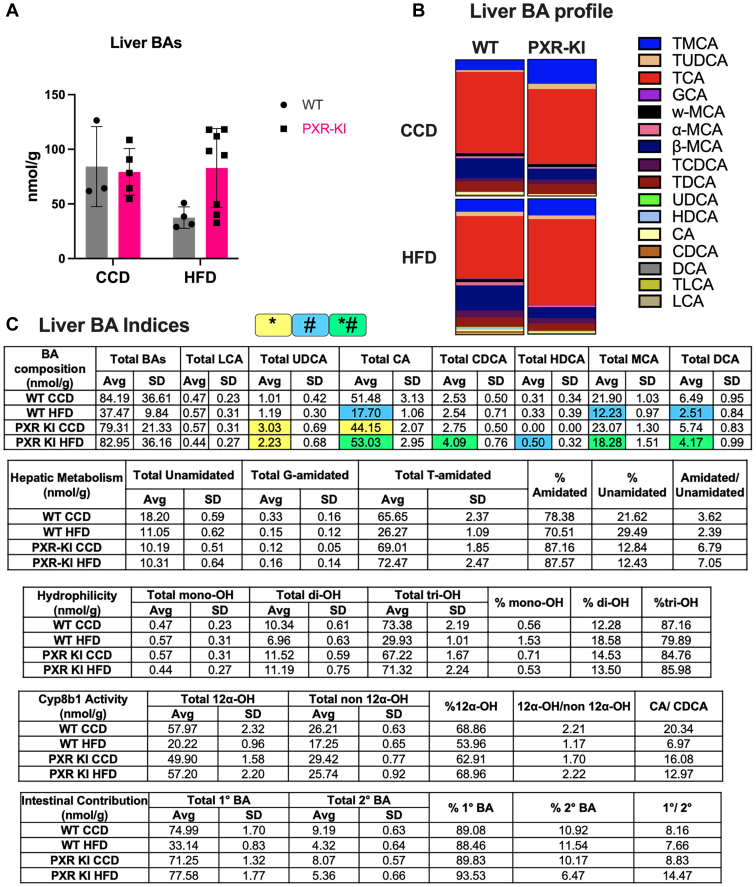
Liver BA profiles and indices. (A) Liver BA concentration. (B) Liver BA individual species as the percentage of total BA pool per treatment group. (C) Liver BA indices calculations for the following criteria: BA composition, hepatic metabolism, hydrophilicity, Cyp8b1 activity, and intestinal absorption. Data in the table are presented as mean ± SD (*n* = 3–8). Two-way ANOVA. ∗ represents the significant difference between genotypes within the same diet, # represents the significant difference between diets within the same genotype, and ∗# represents the significant difference across both genotypes and diets. BA, bile acid; CA, cholic acid; CCD, control chow diet; CDCA, chenodeoxycholic acid; DCA, deoxycholic acid; GCA, glychcholic acid; HDCA, hyodeoxycholic acid; HFD, high-fat diet; LCA, lithocholic acid; MCA, muricholic acid; MDCA, murideooxycholic acid; PXR-KI, pregnane X receptor Ser347Ala knock-in mutation; TCA, taurocholic acid; TCDCA, taurochenodeoxycholic acid; TDCA, taurodeoxycholic acid; TLCA, taurolithocholic acid; TMCA, tauromuricholic acid; TUDCA, tauroursodeoxycholic acid; UDCA, ursodeoxycholic acid; WT, wild-type.

In the liver, WT mice on HFD showed a decrease in total BA concentration, an increase in TMCA (9.21%), *β*-MCA (18.68%), TUDCA (3.17%), and TCDCA (5.11%), but a decrease in TCA (45.76%) compared with the WT CCD group (Fig. 6, A and B). In PXR-KI mice, compared with CCD, HFD feeding resulted in increased TCA percentages (62.99%) and decreased percentages of TMCA (12.26%), TUDCA (2.68%), and *ω*-MCA (0.31%) (Fig. 6B). PXR-KI mice on HFD compared with WT on HFD showed a higher total BA concentration, and the BA pool demonstrated elevated levels of TMCA (12.26%) and TCA (62.99%), but decreased levels of *α*- and *β*-MCA (1.19% and 8.27%) (Fig. 6, A and B). The BA pool also contained more conjugated BA, higher percentages of tri-OH BA shifting the pool toward a more hydrophilic state, an elevated 12*α*-OH/non–12*α*-OH ratio, and increased primary BA/secondary BA (Fig. 6C).

**Fig. 7:**
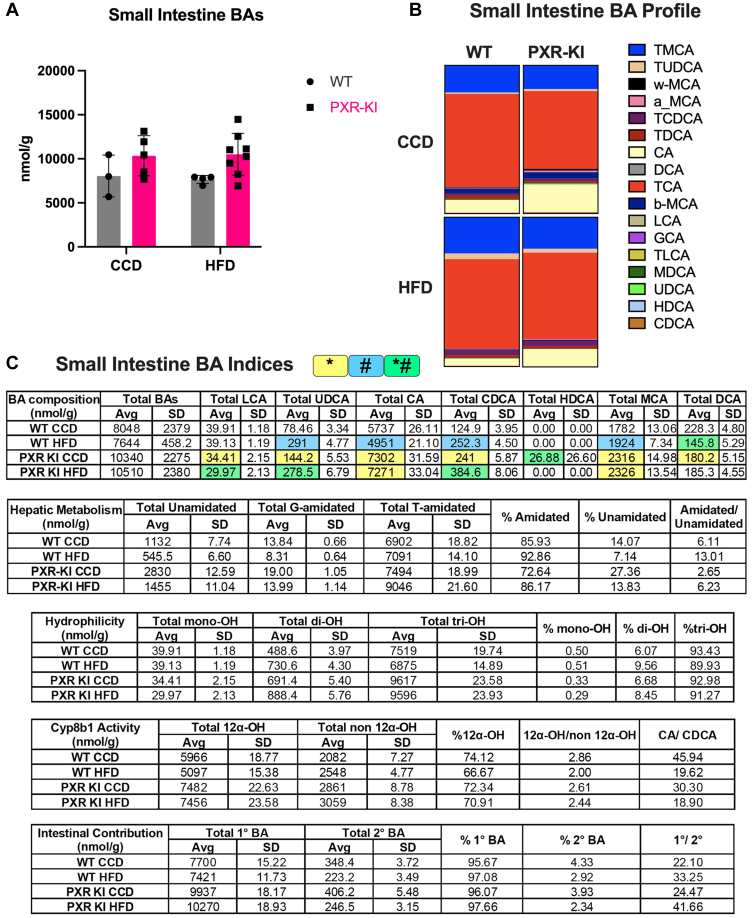
Small intestine BA profiles and indices. (A) Small intestine BA concentration. (B) Small intestine BA individual species as the percentage of total BA pool per treatment group. (C) Small intestine BA indices calculations for the following criteria: BA composition, hepatic metabolism, hydrophilicity, Cyp8b1 activity, and intestinal absorption. Data in the table are presented as mean ± SD (*n* = 3–8). Two-way ANOVA. ∗ represents the significant difference between genotypes within the same diet, # represents the significant difference between diets within the same genotype, and ∗# represents the significant difference across both genotypes and diets. BA, bile acid; CA, cholic acid; CCD, control chow diet; CDCA, chenodeoxycholic acid; DCA, deoxycholic acid; GCA, glychcholic acid; HDCA, hyodeoxycholic acid; HFD, high-fat diet; LCA, lithocholic acid; MCA, muricholic acid; MDCA, murideooxycholic acid; PXR-KI, pregnane X receptor Ser347Ala knock-in mutation; TCA, taurocholic acid; TCDCA, taurochenodeoxycholic acid; TDCA, taurodeoxycholic acid; TLCA, taurolithocholic acid; TMCA, tauromuricholic acid; TUDCA, tauroursodeoxycholic acid; UDCA, ursodeoxycholic acid; WT, wild-type.

In the small intestine, the total BA concentration was higher at the basal level in PXR-KI mice on CCD and PXR-KI mice on HFD (not significant). In PXR-KI mice, compared with CCD, HFD feeding resulted in increased percentages of TMCA (20.91%), TUDCA (2.65%), and TCDCA (3.39%), and decreased percentages of *ω*-MCA (0%) and *α*- and *β*-MCA (0.43%, 0.77%) (Fig. 7, A and B). Compared with WT mice on HFD, PXR-KI mice on HFD showed a decrease in percentages of TMCA (20.91%) and TUDCA (2.65%), but increased CA (11.56%) percentage (Fig. 7B). The BA pool of PXR-KI mice on HFD contained more unconjugated BA compared with WT mice on HFD and less unconjugated BA compared with the PXR-KI CCD group. Compared with WT mice on HFD, the percentage of tri-OH was slightly elevated in PXR-KI mice on HFD, indicating a slight shift toward a more hydrophilic BA pool. Among all groups, PXR-KI mice on HFD demonstrated the highest primary to secondary BA ratio (Fig. 7C). The limit of quantification (LOQ) of all bile acids in serum, liver, and intestine are listed in Supplemental Table 3.

## 4 Discussion

PXR regulation of endobiotic metabolism through post-translational modifications, such as phosphorylation, remains unclear. This study is the first to use the PXR-KI mouse model to investigate the role of blocking PXR phosphorylation at Ser347 in mice on MASH progression. PXR-KI mice on HFD showed increased serum cholesterol and higher LW/BW% (Fig. 1). These mice also exhibited an upregulation of genes associated with hepatic steatosis (Fig. 2) and alterations in BA-related gene expression and profiling compared with WT on HFD (Fig. 3, Fig. 4, Fig. 5, Fig. 6, Fig. 7). These data suggest that PXR-KI mice are more susceptible to HFD-induced liver lipid accumulation and may experience accelerated progression toward MASH.

PXR-KI mice showed increased hepatic de novo lipogenesis and fatty acid uptake. The PPAR*γ* is a positive regulator of lipogenic gene expression, including *Cd36*, *Fasn*, and *Fsp27*, promoting lipid uptake, triglyceride accumulation, and storage, all contributing to fatty liver generation.^22^ CD36 facilitates free fatty acid uptake into the hepatocytes, and its upregulation has been associated with increased hepatic steatosis and insulin resistance in patients with MASH and HFD-fed mice.^23^^,^^24^ Moreover, the overexpression of *Fsp27*, target gene of PPAR*γ*, is associated with increased hepatic steatosis, suggesting its role in promoting lipid droplet formation and triglyceride storage in the liver.^25^ In our study, PXR-KI mice on HFD showed an increase in mRNA levels of *Cd36, Fasn,* and *Fsp27* compared with WT mice on HFD. Also, *Cyp4a10* expression involved in fatty acid *β*-oxidation was elevated by HFD feeding in PXR-KI mice compared with WT mice (Fig. 2A). These data indicate that this group of mice had more lipid uptake, deposition, and storage, which contributed to severe hepatic steatosis (Fig. 1C) that, subsequently, can lead to accelerated MASH disease progression.

To further characterize the impact of blocking PXR at Ser347 on MASH disease development, we evaluated key disease hallmarks, including inflammation and fibrosis. Lcn2, an acute-phase protein in inflammatory response, was reported to be upregulated in the liver and serum of both patients with MASH and mice models.^26^^,^^27^ Interestingly, Lcn2 is also a direct PXR target gene.^28^ In our study, the *Lcn2* expression was upregulated in both WT and PXR-KI mice on HFD, with higher mean levels in PXR-KI mice, suggesting advanced progression to steatohepatitis (Fig. 2B). However, no changes were observed in F4/80 immunostaining (Supplemental Fig. 3A). Also, we did not observe fibrosis development in this model, likely due to 16 weeks of feeding was insufficient for detectable protein level changes despite gene-level alterations (Fig. 2C).^29^^,^^30^

BAs are liver-derived signaling molecules essential for intestinal fat absorption. BA synthesis occurs via 2 pathways: the classical pathway, producing ∼90% of BAs in humans and ∼75% in mice, and the alternative pathway, accounting for the remainder.^31^ Disruption of BA homeostasis is associated with MASH pathogenesis.^21^ BAs regulate their synthesis primarily through ileal FXR activation, which induces FGF19 in human and Fgf15 in mice, leading to activation of the mitogen-activated protein kinase pathway to suppress *CYP7A1/Cyp7a1* and *CYP8B1/Cyp8b1* expression, key enzymes in BA synthesis.^32^^,^^33^ PXR also regulates BA synthesis, conjugation, and efflux processes. Intestinal PXR activation induced FGF19 production, and the FGF19 promoter contains a PXR response element.^34^^,^^35^ However, conflicting reports showed decreased *Fgf15* expression in pregnenolone-16-*α*-carbonitrile-treated WT mice, and increased *Fgf15* in PXR-KO mice fed a lithogenic diet or HFD, contributing to improved HFD-induced obesity.^36^^,^^37^ These findings suggest that PXR may interact with FXR through the *FGF15/19-CYP7A1* axis.^6^ Therefore, we further investigated the impact of blocking PXR phosphorylation at ser347 on BA homeostasis. In PXR-KI mice on HFD, the mean relative mRNA levels of *Cyp7a1*, a classical BA synthesis gene, were reduced, likely because of increased ileal *Fgf15* expression (Fig. 3C), and were consistent with decreased C4 serum levels, a CYP7A1 activity marker (Fig. 5A). In addition, PXR-KI mice on HFD also showed the lowest expression of *Cyp7b1* gene involved in alternative pathways of BA synthesis (Fig. 3A), aligning with previous reports of reduced *Cyp7b1* expression in both patients with MASH and MASH mouse models.^38^ This further indicates that PXR-KI mice on HFD exhibited disrupted BA homeostasis.

In mice, CYP2C70 converts the hydrophobic BA, CDCA, into muricholic MCAs, which are more hydrophilic. Similar to *Cyp7b1, Cyp2c70* expression was reduced at basal expression in PXR-KI mice and further downregulated after HFD feeding. PXR-KI mice on HFD exhibited the lowest expression of both *Cyp2c70* and *Cyp7b1* across all groups (Fig. 3, A and B). The alternative BA synthesis pathway has a beneficial effect on glucose and lipid metabolism.^39^ The shift from the classical to the alternative pathway results in the production of more hydrophilic BAs in mice, which reduces intestinal fat and cholesterol absorption.^40^ Thus, suppression of the alternative pathway in PXR-KI mice likely contributes to increased intestinal cholesterol and fat absorption and hepatic lipid accumulation.

Given the observed changes in BA synthesis and metabolism, we examined ileal and hepatic FXR activity in PXR-KI mice on HFD and observed increased ileal FXR activation, indicated by *Fgf15* induction (Fig. 3C), whereas hepatic FXR activation was reduced corresponding to decreased lipocalin 13 (*Lcn13*) expression (Supplemental Fig. 2C). Notably, FXR plays a crucial role in preventing lipid accumulation, exerting anti-inflammatory effects, and protecting against fibrosis. Consistent with our results, Lcn13 downregulation has been reported in MASH animal models, further exacerbating hepatic steatosis and the inflammatory response.^41^ The data suggest that blocking PXR phosphorylation at Ser347 impairs hepatic FXR activity, contributing to MASH development and highlighting a potential crosstalk between PXR and FXR signaling.

To further evaluate potential cholestasis or alterations in BA flow, we determined hepatic and ileal transporters involved in enterohepatic circulation. During liver injury, the liver uses protective mechanisms by decreasing BA uptake through the downregulation of sodium taurocholate cotransporting polypeptide (NTCP) and organic anion transporting polypeptide (OATP) uptake transporters to limit further damage.^42^ Indeed, the PXR-KI mice on HFD showed a downregulated expression of BA uptake transporter *Oatp1a1* and BA export transporter *Bsep* (Fig. 4A). Others also reported a remarkable decrease in *Bsep* expression in their MASH mouse model.^43^ These changes in PXR-KI mice on HFD suggest intrahepatic BA accumulation, indicative of cholestasis. Overall, BA synthesis, metabolism, conjugation, and signaling pathways were altered in the PXR-KI mice group after 16 weeks of HFD feeding. This could lead to more toxic BA buildup, which can further contribute to more severe liver damage in this group of mice.

To understand the impact of PXRSer347A on the BA pool and composition after all the gene expression changes, we performed BA profiling for serum, liver, and small intestine tissues. PXR-KI mice on HFD showed higher mean levels of total BA concentration in the serum and liver compared with WT mice on HFD (Figs. 5 and 6A), consistent with our previous observation of cholestatic liver, suggesting a coherent pattern of impaired BA flow, hepatic BA retention, and exacerbated liver injury. BA profiling revealed that the serum and hepatic BA pool of PXR-KI mice on HFD compared with WT mice on HFD had more conjugated BA, higher TCA levels, elevated levels of primary BA, a greater percentage of tri-OH BA, and an elevated 12*α*-OH/non–12*α*-OH ratio, indicating increased CYP8B1 activity and a shift toward a more hydrophilic BA pool (Figs. 5 and 6C). In the small intestine, BA pool of PXR-KI mice contained more unconjugated BA, a higher percentage of tri-OH, and the highest primary to secondary BA ratio (Fig. 7C). Our serum and liver findings align with previous studies reporting elevated conjugated and primary BA, as well as an increased CA/CDCA ratio in patients with MASH. In addition, elevated TCA levels have been linked to greater steatosis severity and increased hepatocyte ballooning in patients with MASH.^44^^,^^45^

Notably, the elevated hepatic and serum 12*α*-OH/non–12*α*-OH ratio in PXR-KI mice on HFD could be due to increased TCA production via the classical pathways and reduced non–12-hydroxylated BA from downregulated alternative pathways. Interestingly, a higher ratio of 12-OH (CA)/non–12-OH (CDCA) has been associated with hepatic steatosis in HFD-fed rats^46^ and insulin resistance in humans.^47^ Overall, PXR-KI mice showed more pronounced changes in the expression of genes associated with lipid and BA metabolism after 16 weeks of HFD feeding, which indicates the important role of PXR phosphorylation at Ser347 as a physiological sensor in maintaining energy homeostasis and endobiotic metabolism.

## 5 Conclusion

This work investigates the role of phosphorylating PXR at Ser347 in maintaining endobiotic metabolism during MASH development. Our findings indicate that blocking PXR phosphorylation at Ser347 in mice altered lipid and BA homeostasis. These alterations can promote hepatocellular stress, inflammation, and fibrosis, thereby exacerbating MASH disease progression over time. This study implies that PXR phosphorylation at Ser347 in mice plays an important role in regulating endobiotic metabolism and mitigating liver damage and disease development. These results pave the way for further research to uncover the underlying molecular mechanisms by which phosphorylated PXR at Ser347/350 regulates endobiotic metabolism during MASH development, particularly in crosstalk with other nuclear receptors for the regulation of lipid and BA homeostasis. Understanding these mechanisms could also be valuable for drug discovery and identifying new intervention targets to prevent or treat MASH.

## Conflict of interest

The authors declare no conflicts of interest.
