# Supplementary material for: Mutation of PXR phosphorylation motif at Ser347 disrupts lipid and bile acid homeostasis in diet-induced metabolic dysfunction–associated steatohepatitis in mice
Source: Drug Metab Dispos. 2025 Dec 22;54(2):100222. doi: 10.1016/j.dmd.2025.100222 (PMC12975368; doi:10.1016/j.dmd.2025.100222)
Supplement: Supplementary Table 1-3 and Supplementary Figure 1-4 [file mmc1.pdf]

Supplemental Tables and Figures

Supplemental Table 1. Primer Sequences

Primer	Forward	Reverse
α-Sma	CCTGACGGGCAGGTGATC	ATGAAAGATGGCTGGAAGAGAGTCT
Amacr	GTGGATGAACAGCAATGAAGTCA	CGCAATCGTTATCCTGTAACCA
Asbt	TTGCACAGCACAAGCAGTGA	TGCATTGAAGTTGCTCTCAGGT
Baat	AGCACCACTCCTCACTTCCATAG	TCCATCCTCCTGTATTTTCTTGTG
Bsep	GCAGAAGCAAAGGGTAGCCATC	GGTAGCCATGTCCAGAAGCAG
Cd36	GATGACGTGGCAAAGAACAG	TCCTCGGGGTCCTGAGTTAT
Col1a1	GAGAGAGCATGACCGATGGATT	TGTAGGCTACGCTGTTCTTGCA
Cyp3a11	ACAAGGGTTTATGGAAATTCG	GTCTGTGACAGCAAGGAGAGG
Cyp27a1	GCTTTCTCTTCCCCAAG	CAGCCTCTTTCTTCCTCA
Cyp2b10	GACTTTGGGATGGGAAAGAG	CCAAACACAATGGAGCAGAT
Cyp2b9	TGGCCACCATGAAAGAGTTTG	GCTGTGATGCACTGGAAGAGAA
Cyp2c70	TGGCTTTCTCAGCAGGAAGAA	AACTGGCTTGGTGTCGATGT
Cyp4a10	TTCCCTGATGGACGCTCTTTA	GCAAACCTGGAAGGGTCAAAC
Cyp7a1	AGCAACTAAACAACCTGCCAGTACTA	GTCCGGATATTCAAGGATGCA
Cyp7b1	CAGCTATGTTCTGGGCAATG	TCGGATGATGCTGGAGTATG
Cyp8b1	AGTACACATGGACCCCGACATC	GGGTGCCATCCGGGTTGAG
Fasn	GCTGCGGAAACTTCAGGAAAT	AGAGACGTGTCACTCCTGGACTT
Fgf15	GAGGACCAAAACGAACGAAATT	ACGTCCTTGATGGCAATCG
Fgf21	ATCAGGGAGGATGGAACAGTGG	AGCTCCATCTGGCTGTTGGCAA
Fsp27	GCCACAGCACCAACTATGTC	ACACTCTCTCGCACACCTCA
G6Pase	CCGGATCTACCTTGCTGCTCACTTT	TAGCAGGTAGAATCCAAGCGCGAAAC
Ibabp	GGTCTTCCAGGAGACGTGAT	ACATTCTTTGCCAATGGTGA

Il-1β	AAGGGCTGCTTCCAAACCTTTGAC	ATACTGCCTGCCTGAAGCTCTTGT
IL-6	ATCCAGTTGCCTTCTTGGGACTGA	TAAGCCTCCGACTTGTGAAGTGGT
Lcn13	ACAATGGTACCTACCCAGTCACA	ACTCACGGCAATGACCATTGTTCC
Lcn2	AATGTCACCTCCATCCTGGTCA	CCACTTGCACATTGTAGCTCT
Mrp2	ACTGGACAAGCCACAATTCC	CTGCAGGAGTGCTCGTATCA
Mrp3	CCCTGCGTATGAATTAGTC	CTGCCTCTGGCCAACACTG
Mrp4	TTAGATGGGCCTCTGGTTCT	GCCCACAATTCCAACCTTT
Ntcp	GGCCACAGACACTGCGCT	AGTGAGCCTTGATCTTGCTGAACT
Oatp1a1	TTCATTTTCACATGGCATTTTCTC	AACACAACTCCCCTTGATTGAGTTA
Oatp1a4	ATAGCTTCAGGCGCATTTAC	TTCTCCATCATTCTGCATCG
Osta	GTCTCAAGTGATGAACTGCCA	TTGAGTGCTGAGTCCAGGTC
Ostb	GTATTTTCGTGCAGAAGATGCG	TTTCTGTTTGCCAGGATGCTC
Pepck	CCACAGCTGCTGCAGAACA	GAAGGGTCGCATGGCAAA
Pparg2	ACTCTGGGAGATTCTCCTGTTG	CCAGAATGGCATCTCTGTGTC
Shp	CGATCCTCTTCAACCCAGATG	AGGGCTCCAAGACTTCACACA
Timp1	GAGACCACCTTATACCAGCGT	CTGGGACTTGTGGGCATATC

Supplemental Table 2. Immunohistochemistry Antibody List

Antibody	Manufacturer	Catalog No.	Dilution
F4/80	Biorad, Hercules, CA	MCA497GA	1/1000
Rat IgG 2b Negative Control	Biorad, Hercules, CA	MCA1125R	1/1000

	Average LOQ Values		
	Serum (nM)	Liver (nmol/g)	Intestine (nmol/g)
**TMCA**	96.96	0.194	9.70
**TUDCA**	100.06	0.400	7.00
**TCA**	290.87	0.388	19.39
**GCA**	NF	0.215	10.74
**ω-MCA**	489.48	0.489	30.59
**α-MCA**	244.74	0.489	18.36
**ß-MCA**	244.74	0.489	36.71
**TCDCA**	100.06	0.400	10.01
**TDCA**	60.03	0.200	5.00
**MDCA**	1018.93	NF	101.89
**UDCA**	382.07	0.509	38.21
**HDCA**	764.18	1.019	50.95
**CA**	734.21	0.245	85.66
**CDCA**	254.73	1.019	25.47
**DCA**	127.37	0.306	12.74
**TLCA**	NF	0.207	5.27
**LCA**	796.60	2.124	66.38
**C4**	1997.00	1.997	199.70

Supplemental Table 3. BA Limit of Quantification (LOQ) Values. The LOQ values were determined for each individual BA species in the serum, liver, and intestine. The LOQs were concentrations with signal-to-noise ratio (S/N) ≥ 3. *NF stands for not found.

A. Glucose Tolerance test

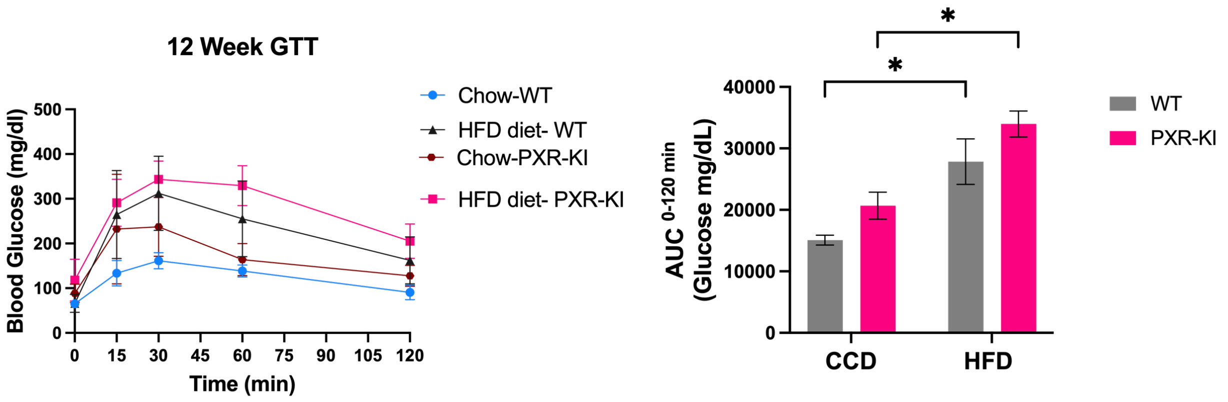

B. Serum Biochemistry

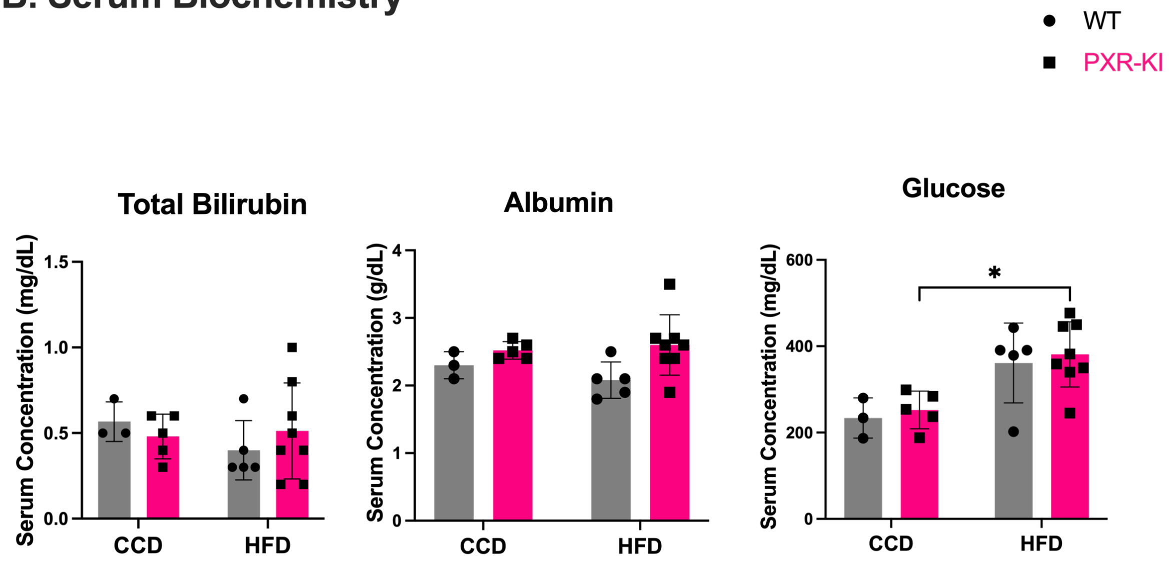

Supplemental Figure 1. Glucose Tolerance Test (GTT) and Serum Biochemistry.

GTT was performed at week 12. A.) Glucose tolerance curves and area under the curve; B.) Serum Albumin, Bilirubin and Glucose. Data represented as the mean ± SD (n = 3-8). Two-way ANOVA. *$p < 0.05$, **$p<0.01$, ***$p<0.001$, ****$p<0.0001$.

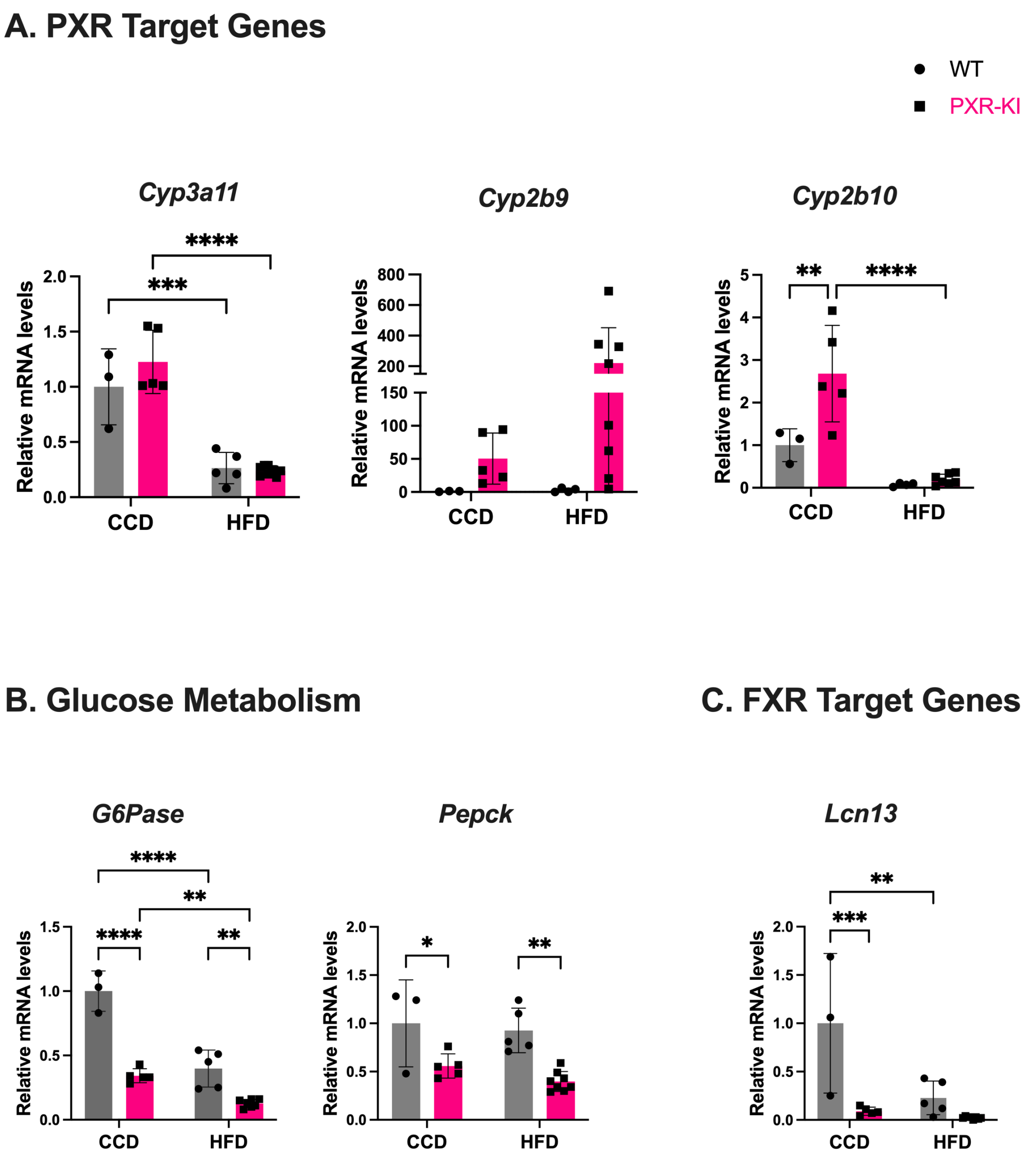

Supplemental Figure 2. Relative mRNA Quantification of PXR Target Genes, Glucose Metabolism, and FXR Target Genes.

A.) mRNA expression of PXR target genes (*Cyp3a11, Cyp2b9, Cyp2b10*); B.) mRNA expression of glucose metabolism related genes (*G6Pase, Pepck*); C.) mRNA expression o FXR target gene (*Lcn13*). Data represented as mean ± SD (n = 3-8). Two-way ANOVA. *p < 0.05, **p<0.01, ***p<0.001, ****p<0.0001.

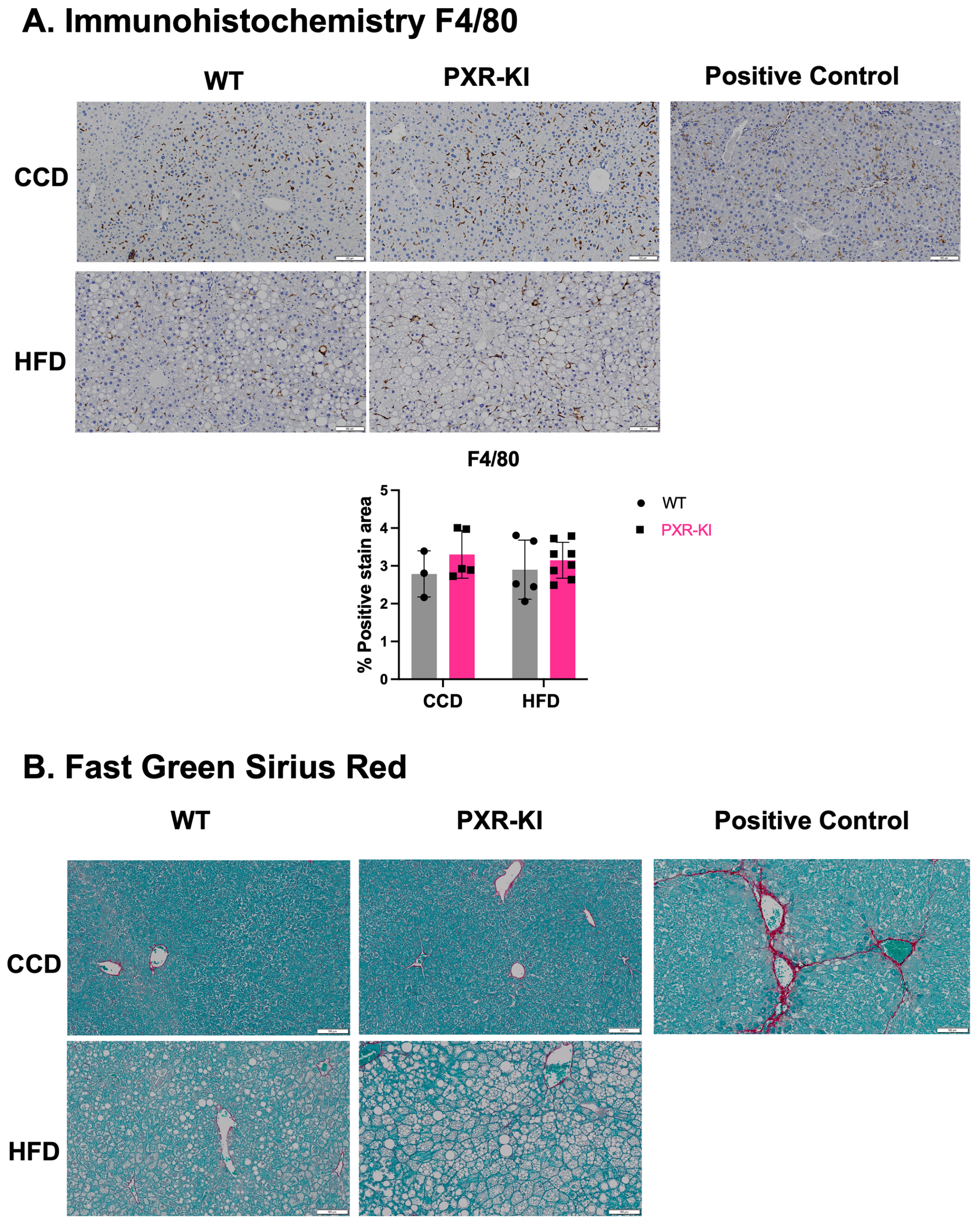

Supplemental Figure 3. Inflammation and Fibrosis.

A.) Immunohistochemistry of liver tissues for F4/80 with corresponding semiquantitative analysis of % positive staining area; B.) Fast green Sirius red staining. Data represented as the average quantification of 5 pictures per animal per treatment group (n = 3-8). Two-way ANOVA. *$p < 0.05$, **$p<0.01$, ***$p<0.001$, ****$p<0.0001$.

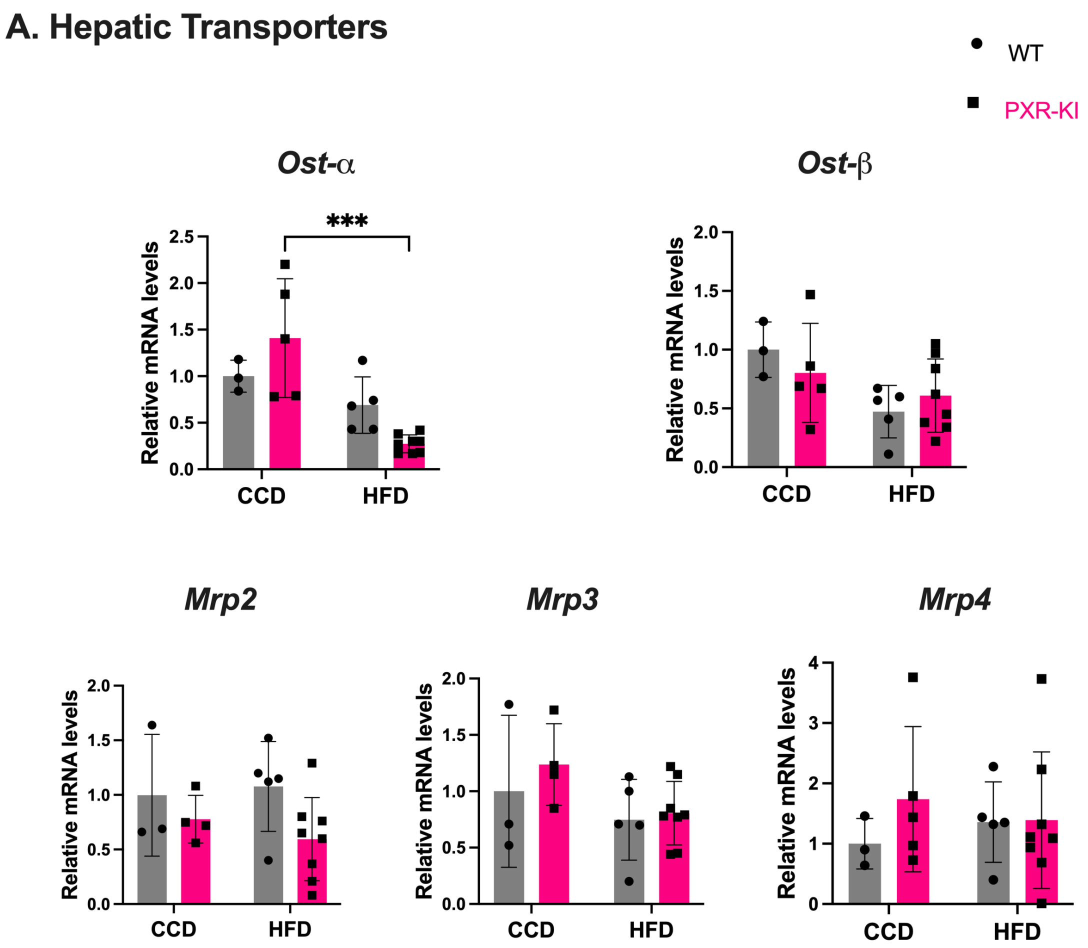

Supplemental Figure 4. Relative mRNA Quantification of Hepatic Transporter Genes.

A.) mRNA expression of hepatic BA transporters (*Ost-α, Ost-β, Mrp2, Mrp3, Mrp4)*; Data represented as mean ± SD (n = 3-8). Two-way ANOVA. *$p < 0.05$, **$p<0.01$, ***$p<0.001$, ****$p<0.0001$.
